# Feasibility Testing Bootle Blast as an Exergame Intervention for People Living with Dementia and Family Caregivers at Home

**DOI:** 10.1002/alz70858_105469

**Published:** 2025-12-26

**Authors:** Erica Dove, Rosalie H. Wang, Kara K. Patterson, Elaine Biddiss, Arlene Astell

**Affiliations:** ^1^ KITE Research Institute, Toronto Rehabilitation Institute, University Health Network, Toronto, ON, Canada; ^2^ University of Toronto, Toronto, ON, Canada; ^3^ Holland Bloorview Kids Rehabilitation Hospital, Toronto, ON, Canada; ^4^ Northumbria University, Newcastle upon Tyne, United Kingdom

## Abstract

**Background:**

Physical exercise can target fall risk factors that affect people living with dementia, such as poor balance and concerns about falling. Existing exercise programs are criticized for lacking engagement and accessibility for people with dementia, warranting more innovative solutions. Bootle Blast, a movement‐tracking video game co‐designed and validated with youth experiencing musculoskeletal disability, includes lower‐body games targeting balance. Preliminary testing by people living with dementia suggests the games may be applicable to them. The current study is testing Bootle Blast for people living with dementia, examining feasibility (i.e., usability, acceptability, safety, and enjoyment).

**Methods:**

This multi‐methods study is recruiting 10 pairs of people with dementia and their family caregivers via convenience sampling in the community. Pairs will play four lower‐body games on Bootle Blast for two weeks at home using a self‐chosen dose. Before and after the intervention, participants will complete outcome measures targeting cognition, concerns about falling, and balance. During the intervention, videos will be collected to capture skeletal/kinematic data (to profile participants’ movement quality) and enjoyment. Participants will also be called weekly to check in on their progress. Post‐study interviews with pairs will capture their enjoyment and perceptions of using Bootle Blast at home.

**Results:**

Results will profile participants’ cognition, balance, movement quality (via skeletal data), and concerns about falling. Post‐study interviews with people with dementia and their family caregivers will provide insight into intervention feasibility and potential alterations that could be made to the games. Recruitment for this project is underway, and results will be ready for presentation at the conference.

**Conclusions:**

Findings from this study will inform future design adaptations based on participants’ feedback regarding the system's usability, acceptability, safety, and enjoyment for people living with dementia at home. The results will also inform a future pilot study of Bootle Blast's impacts on balance and fall risk in a larger sample of people with dementia and family carers.

